# Supplementary material for: Degradation of P(3HB-co-4HB) Films in Simulated Body Fluids
Source: Polymers (Basel). 2022 May 13;14(10):1990. doi: 10.3390/polym14101990 (PMC9143980; doi:10.3390/polym14101990)
Supplement: Supplementary file 1 [file polymers-14-01990-s001.zip › polymers-1693651-supplementary.pdf]

polymers

MDPI

Supplementary material to:

Degradation of P(3HB-*co*-4HB) films in Simulated Body Fluids

Juraj Vodicka[1], Monika Wikarska[1], Monika Trudicova[1], Zuzana Juglova[1], Aneta Pospisilova[1], Michal Kalina[1], Eva Slaninova[1], Stanislav Obruca[1], Petr Sedlacek[1,*]

[1] Faculty of Chemistry, Brno University of Technology, Purkynova 118, 612 00 Brno, Czech Republic
* Corresponding author: Petr Sedlacek, sedlacek-p@fch.vut.cz; Tel.: +420 541 149 486

Table S1: MES composition.

Component	Amount [g/L]
$FeCl_3 \cdot 6\ H_2O$	9.7
$CaCl_2 \cdot 2\ H_2O$	7.8
$CuSO_4 \cdot 5\ H_2O$	0.156
$CoCl_2 \cdot 6\ H_2O$	0.119
$NiCl_2 \cdot 6\ H_2O$	0.118
$CrCl_2$	0.062
0,1 M HCl	1000 mL

Table S2: TES composition.

Component	Amount [g/L]
EDTA	50
$FeCl_3$	8.3
$ZnCl_2$	0.84
$CuCl_2 \cdot 2\ H_2O$	0.13
$CoCl_2 \cdot 6\ H_2O$	0.1
$MnCl_2 \cdot 6\ H_2O$	0.016
$H3BO_3$	0.1
Water	1000 mL

Table S3: Composition of phosphate buffer solution (PBS) for films incubation.

Component	Amount [g/L]
NaCl	8
KCl	0.2
Na_2HPO_4	1.44
KH_2PO_4	0.245
Water	1000 mL

Table S4: Composition of simulated gastric juice (SGJ).

Component	Amount [g/L]
Sodium taurocholate	0.043
Lecithin (soy)	0.002
Pepsin	0.1
NaCl	2.0
Water	1000 mL

Table S5: Composition of artificial colonic fluid (ACF). Tris-maleate buffer consisted of 5.5 g of Tris and 8.8 g o maleic acid, adjusted by 0.5M NaOH to pH 7.8.

**Component**	**Amount [g/L]**
Porcine bile extract	0.113
Lecithin (soy)	0.222
Palmitic acid	0.026
Bovine serum albumin	3
Pancreatin (porcine)	0.1
Tris-maleate buffer	1000 mL

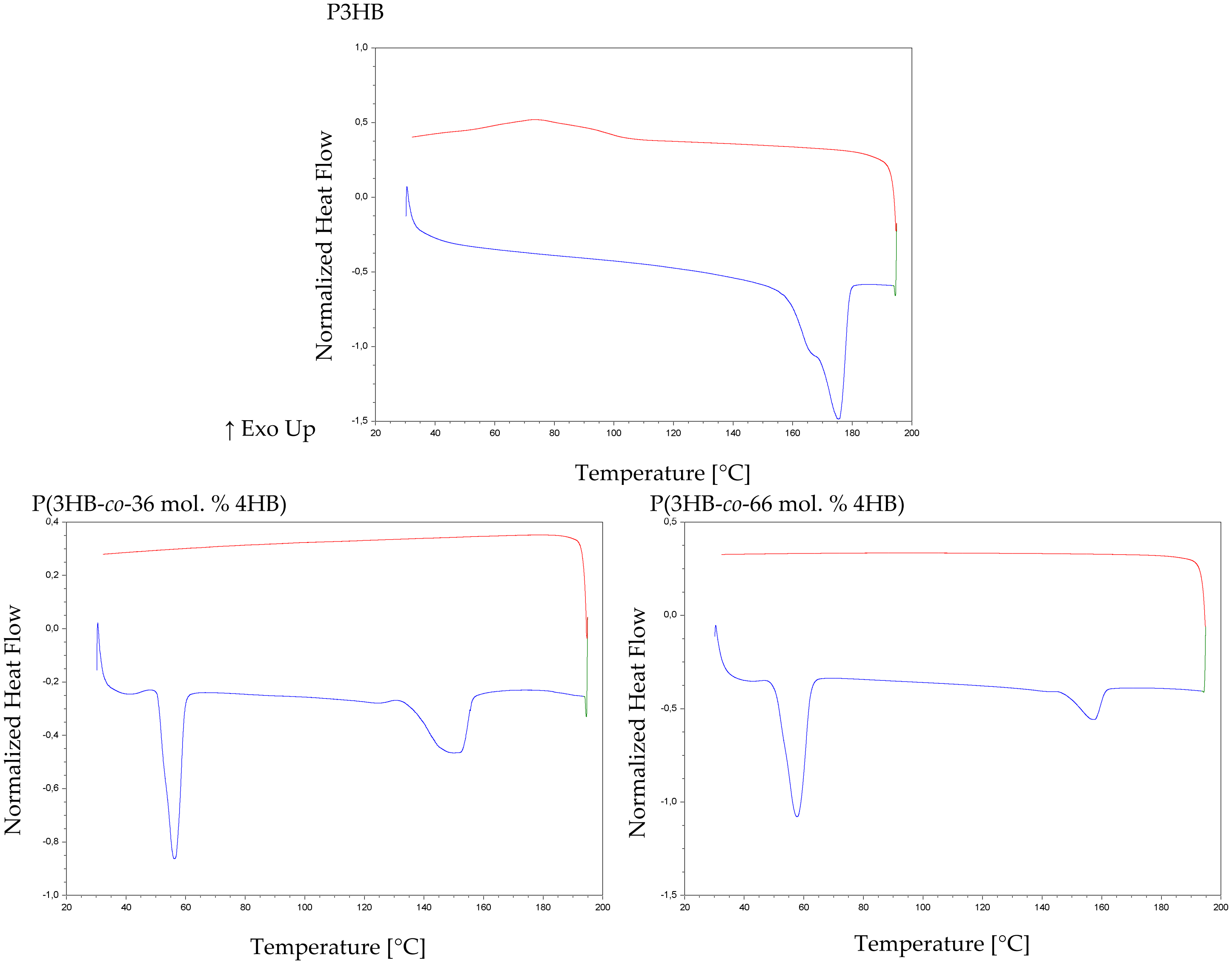

Figure S1: DSC thermograms of initial-state polymer films.

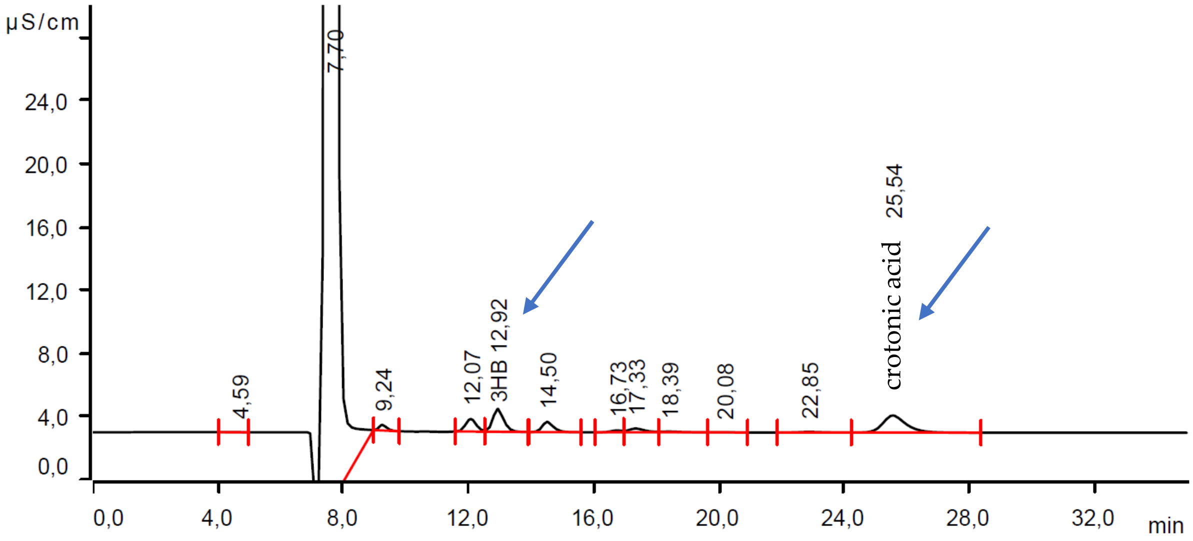

Figure S2: IC chromatogram of pure ACF with 3HB and crotonic acid standards. The arrows point peaks of standards.

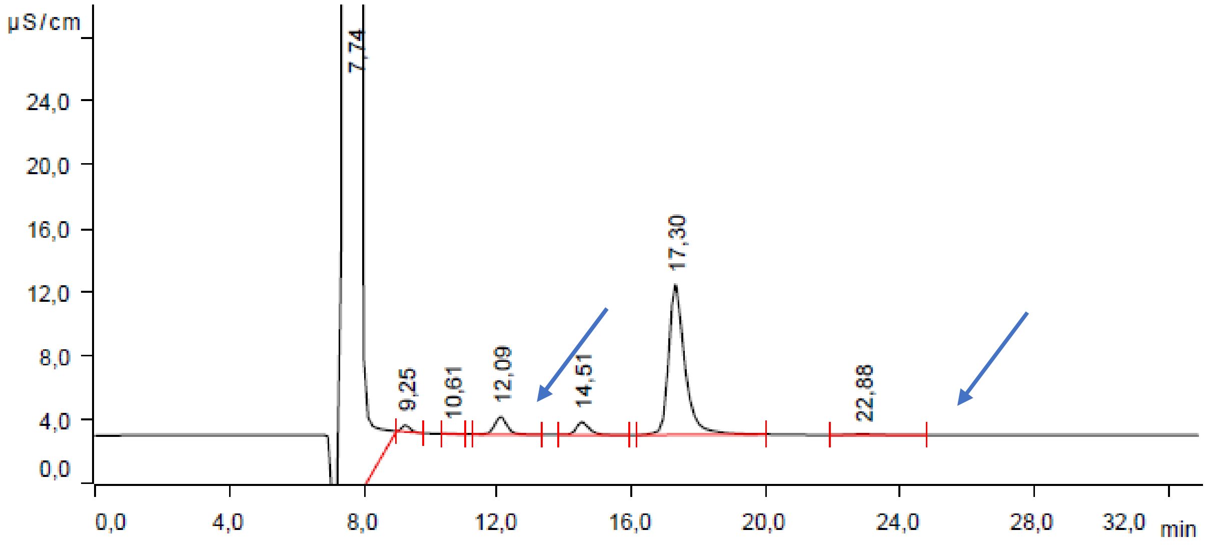

Figure S3: IC chromatogram of waste ACF. The arrows point missing peaks of 3HB and crotonic acid.
